# The effects of kinase modulation on *in vitro* maturation according to different cumulus-oocyte complex morphologies

**DOI:** 10.1371/journal.pone.0205495

**Published:** 2018-10-11

**Authors:** Bong-Seok Song, Pil-Soo Jeong, Jong-Hee Lee, Moon-Hyung Lee, Hae-Jun Yang, Seon-A Choi, Hwal-Yong Lee, Seung-Bin Yoon, Young-Ho Park, Kang-Jin Jeong, Young-Hyun Kim, Yeung Bae Jin, Ji-Su Kim, Bo-Woong Sim, Jae-Won Huh, Sang-Rae Lee, Deog-Bon Koo, Kyu-Tae Chang, Sun-Uk Kim

**Affiliations:** 1 Futuristic Animal Resource & Research Center, Korea Research Institute of Bioscience and Biotechnology, Chungcheongbuk-do, Republic of Korea; 2 National Primate Research Center, Korea Research Institute of Bioscience and Biotechnology, Chungcheongbuk-do, Republic of Korea; 3 Department of Bioscience, University of Science and Technology, Daejeon, Republic of Korea; 4 Department of Biotechnology, College of Engineering, Daegu University, Gyeongbuk, Republic of Korea; 5 Department of Biological Science, College of Natural Sciences, Wonkwang University, Jeollabuk-do, Republic of Korea; 6 Primate Resource Center, Korea Research Institute of Bioscience & Biotechnology, Jeollabuk-do, Republic of Korea; 7 Department of Functional Genomics, University of Science and Technology, Daejeon, Republic of Korea; Institut de Genetique et Developpement de Rennes, FRANCE

## Abstract

Successful production of transgenic pigs requires oocytes with a high developmental competence. However, cumulus–oocyte complexes (COCs) obtained from antral follicles have a heterogeneous morphology. COCs can be classified into one of two classes: class I, with five or more layers of cumulus cells; and class II, with one or two layers of cumulus cells. Activator [e.g., epidermal growth factor (EGF)] or inhibitors (e.g., wortmannin and U0126) are added to modulate kinases in oocytes during meiosis. In the present study, we investigated the effects of kinase modulation on nuclear and cytoplasmic maturation in COCs. Class I COCs showed a significantly higher developmental competence than class II COCs. Moreover, the expression of two kinases, AKT and ERK, differed between class I and class II COCs during *in vitro* maturation (IVM). Initially, inhibition of the PI3K/AKT signaling pathway in class I COCs during early IVM (0–22 h) decreased developmental parameters, such as blastocyst formation rate, blastomere number, and cell survival. Conversely, EGF-mediated AKT activation in class II COCs enhanced developmental capacity. Regarding the MAPK signaling pathway, inhibition of ERK by U0126 in class II COCs during early IVM impaired developmental competence. However, transient treatment with U0126 in class II COCs increased oocyte maturation and AKT activity, improving embryonic development. Additionally, western blotting showed that inhibition of ERK activity negatively regulated the AKT signaling pathway, indicative of a relationship between AKT and MAPK signaling in the process underlying meiotic progression in pigs. These findings may help increase the developmental competence and utilization rate of pig COCs with regard to the production of transgenic pigs and improve our understanding of kinase-associated meiosis events.

## Introduction

Transgenic pigs are regarded as potentially good animal models for biomedical research. However, to produce transgenic pigs, porcine cumulus–oocyte complexes (COCs) with high developmental competence are needed because of the low development rate of *in vitro*-produced (IVP) blastocysts compared with embryos produced *in vivo*. Many laboratories have cultured COCs of heterogonous morphology *in vitro* to produce mature oocytes upon fertilization or parthenogenesis (PA) [1, 2]. Moreover, many researchers have cultured COCs to produce IVP embryos according to the number of cumulus cell layers [3, 4]. Several studies have reported on the relationship between follicle diameter and oocyte developmental capacity and on the correlation between oocyte size and developmental competence [1, 5, 6]. Furthermore, researchers have focused on enhancing developmental competence via treatment with supplements, such as growth factors, hormones, and inhibitors, to alter the activity of proteins in COCs [7, 8]. However, there is limited information on the improvement of maturation competence via the regulation of proteins in oocytes according to differences in COC morphology.

The protein kinase B/AKT is a serine/threonine (Ser/Thr) kinase that acts as an intermediate signaling component of the phosphatidylinositol-3-kinase/PKB (PI3K/AKT) pathway. The AKT protein is phosphorylated at two sites, a threonine residue (Thr308) in the catalytic domain and a serine reside (Ser473) at the C-terminus; it transduces intracellular signals by phosphorylating target proteins. In somatic cells, the PI3K/AKT pathway is activated by insulin, growth factors, and adhesion to the extracellular matrix, which promotes cell survival and participates in the regulation of the G2/M transition. In mammalian oocytes, AKT signaling is involved in meiotic progression and is constantly detected in both oocytes and cumulus cells during *in vitro* maturation (IVM) [9, 10]. Although AKT activity is not essential for induction of germinal vesicle breakdown (GVBD), it is required for the transition from metaphase I (MI) to metaphase II (MII) during meiosis [11]. In a previous report, the PI3K/AKT signaling pathway was associated with the developmental competence of bovine oocytes [12]. Additionally, supplementation with certain proteins (e.g., hormones and gangliosides) in IVM medium affects the developmental capacity via modulation of the PI3K/AKT signaling pathway [13, 14]. Therefore, it has been suggested that the AKT activity in oocytes differs between class I and class II COCs and that it might be regulated to increase maturation competence and subsequent early embryonic development.

The extracellular signal-regulated kinase-1/2 (ERK1/2) is a member of the mitogen-activated protein kinase (MAPK) superfamily, which has critical roles in meiotic processes [15]. ERK1/2 is Ser/Thr protein kinases activated by MAPK kinase (MEK)-dependent phosphorylation of both tyrosine and threonine residues [16]. ERK1/2 is a key signaling molecule during meiosis that affects spindle assembly and microtubule organization in oocytes [17]. During meiosis, ERK1/2 is activated around the GVBD stage and peak during MII [18]. They participate in cumulus cell expansion due to the suppression of the expression of expansion-related genes via ERK signaling inhibitors, and inhibition impairs zygotic genome activation, fertilization, and the cleavage rate [19]. We previously reported that culturing COCs with hormones during IVM increased ERK expression during the MII stage and resulted in significantly increased blastocyst development [8, 20]. Additionally, ERK1/2 affectedcytoplasmic maturation, subsequent cleavage, and the blastocyst formation rate of sheep oocytes [21]. Although MAPKs have been reported as essential maturation factors during meiotic events, the relationship between kinase expression and developmental competence in oocytes derived from class I and class II COCs during IVM has not been determined in the pig.

Studies have shown that the PI3K/AKT and ERK1/2 signaling pathways can regulate meiotic events and affect maturation competence in oocytes. However, there is little information on kinase expression and its relationship to the developmental competence of oocytes according to COC class in the pig. Therefore, in this study, we investigated the effect of kinase modulation on the IVM of oocytes derived from class I and class II COCs and increased the developmental competence and utilization rate to yield IVP embryos. We founded that the expression of two kinases, AKT and ERK1/2, showed differences between class I and class II oocytes during IVM. Next, we added an activator (EGF) or inhibitors (wortmannin and U0126) of the signaling pathway to the early maturation culture medium to examine the effects of each on oocyte maturation and developmental capacity. These findings may increase the developmental competence and utilization rate of pig COCs with regard to the production of transgenic pigs and may improve our understanding of kinase-associated meiosis events.

## Materials and methods

### Ethics statement

All procedures and use of pigs were approved by the Korea Research Institute of Bioscience and Biotechnology (KRIBB) Institutional Animal Care and Use Committee (Approcal No.KRIBB-AEC-18098)

### Chemicals

Unless otherwise noted, all chemicals and reagents used in this study were purchased from Sigma-Aldrich Chemical Co. (St. Louis, MO, USA).

### Oocyte collection, classification, and IVM

**Fig 1:**
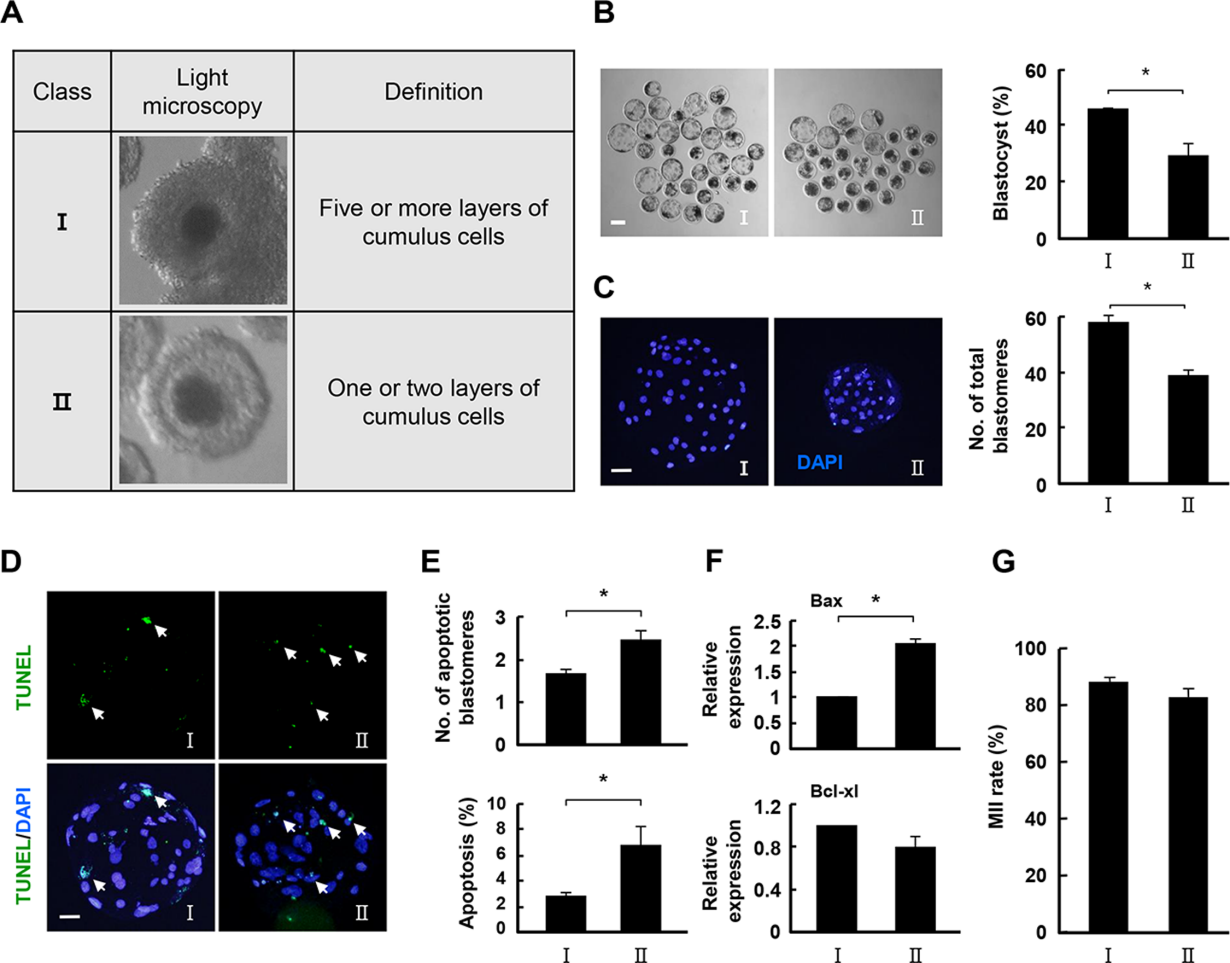
Comparison of the developmental competence in class I and class II COCs. (A) Representative photographs of classified porcine COCs. (B) Representative photographs of developed blastocysts from the indicated classes. Bar = 50 μm. Blastocyst development rate (right) for the indicated classes. The data are from three independent experiments, and the values represent the means ± SEM (*P < 0.05). (C) Nuclear staining of blastomeres using blastocysts cultured from the indicated classes. An image of DAPI (blue) signals is shown. Bar = 50 μm. Quantification of the total number of cells in the indicated classes (right). The data are from three independent experiments, and the values represent the means ± SEM (*P < 0.05). (D and E) Apoptosis detection analysis in blastocysts from the indicated classes. Merged images (light green) between TUNEL (green, white arrow) and DAPI (blue) signals are shown. Bar = 50 μm. Quantification of the number (E, upper) and proportion (E, lower) of apoptotic cells in the indicated classes. The data are from three independent experiments, and the values represent the means ± SEM (*P < 0.05). (F) Relative mRNA expression levels of Bax and Bcl-XL in blastocysts from the indicated classes. The data are from three independent experiments, and the values represent the means ± SEM (*P < 0.05). (G) Nuclear maturation rate of mature oocytes from the indicated classes.

The procedures used for the collection and maturation of COCs in the present study were performed as described previously [22]. Briefly, ovaries were collected from a local slaughterhouse and then transported to the laboratory in a solution of 0.9% saline supplemented with 75 μg/mL potassium penicillin G and 50 μg/mL streptomycin sulfate at 25–30°C within 2 h. Disposable 10 mL syringes with 18-gauge needles were used for COC aspiration from the follicles (2–8 mm in diameter) and then the collected COCs were rinsed three times in Tyrode’s Albumin Lactate Pyruvate-HEPES medium [23]. Each COC was categorized into one of the two classes according to the number of cumulus cell layers (class I COCs: ≥ 5 layers of cumulus cells; class II COCs: 1–2 layers of cumulus cells; Fig 1A) and then cultured in a modified IVM medium, as previously described [22]. Approximately 50 classified COCs were sequentially matured in IVM medium (500 μL) in a four-well multi-dish (Nunc, Roskilde, Denmark) for 44 h in an atmosphere containing 5% CO_2_ at 38.5°C. The early IVM medium (0–22 h) consisted of tissue culture medium 199 supplemented with 10% porcine follicular fluid, 0.57 mM cysteine, 10 ng/mL β-mercaptoethanol, 10 IU/mL pregnant mare serum gonadotropin, and 10 IU/mL human chorionic gonadotropin. After 22 h in the early IVM, the COCs were maintained without hormones for another 22 h. After 44 h of IVM, expanded cumulus cells with oocytes were treated with 0.1% hyaluronidase, vortexed for 1 min for removal, and then washed with DPBS containing 0.4% BSA. Matured MII oocytes with a visible polar body and regular morphology were used for PA and SCNT.

### Chemical treatment

After classification, the class I and class II COCs were cultured in early IVM medium in the presence of either EGF (10 ng/mL; E9644), wortmannin (10 μM; W1628), or U0126 (10 μM, 9903; Cell Signaling Technology, Danvers, MA, USA) in an atmosphere with 5% CO_2_ at 38.5°C. The doses of wortmannin and U0126 were chosen based on previous reports [24–27]. Transient inhibition of MAPK signaling occurred from 17–22 h during early IVM in the presence of U0126.

### PA and IVC

The matured oocytes derived from each group were placed in a 1-mm gab wire chamber (CUY 5000P1; Nepa Gene, Chiba, Japan) overlaid with 10 μL of 280 mM mannitol containing 0.1 mM MgSO4 7H2O, 0.1 mM CaCl2 2H2O, 0.5 mM HEPES, and 0.01% polyvinyl alcohol (PVA) [28]. To prevent variation in oocyte maturation due to sperm factors associated with in vitro fertilization (IVF), an electrical pulse was employed to activate oocyte complementation. PA and IVC were carried out as described previously [22]. The oocytes were activated with one direct current pulse (1.8 kV/cm for 50 μs) provided by an Electro Cell Fusion Generator (LF101; Nepa Gene). Then, the stimulated oocytes were cultured in activation medium (IVC medium supplemented with 5 μg/mL cytochalasin B and 2 mM 6-dimethylaminopurine) for 4 h in an atmosphere with 5% CO_2_ at 38.5°C to induce activation. Following activation, the oocytes were washed in IVC medium, and cultured in IVC medium for 6 days in an atmosphere with 5% CO_2_ at 38.5°C. The developmental parameters were scored on Day 6 of IVC.

### Somatic cell nuclear transfer (SCNT)

Somatic cell nuclear transfer (SCNT) and preparation of donor cells was carried out as described previously [22]; matured oocytes with a visible first polar body were selected for SCNT. Using an automated inverted microscope (DMI 6000B; Leica, Wetzlar, Germany) equipped with a micromanipulator (NT-88-V3; Nikon-Narishige, Tokyo, Japan), denuded oocytes were placed in DPBS supplemented with 4 mg/mL BSA, 75 μg/mL penicillin G, 50 μg/mL streptomycin sulfate, and 7.5 μg/mL CB, and then penetrated by cutting the pipette to make a slit to squeeze approximately 10% of the cytoplasm and the first polar body out of the oocytes. KSP minipig donor cells that were round in shape and 15–20 μm in diameter were selected, and inserted into the perivitelline space through the near slit in the zona pellucida that was made during enucleation. Oocyte-cell couplets were maintained in IVC medium in an atmosphere of 5% CO_2_ at 38.5°C until electrical fusion. For cell fusion, a single oocyte-cell couplet was equilibrated in fusion medium with 280 mM mannitol containing 0.1 mM MgSO4∙7H2O and 0.01% PVA, placed between two parallel electrodes (100 μm in diameter; CUY 5100–100; Nepa Gene) attached to the micromanipulator, rearranged in a straight line, and then activated by a single direct current pulse (23 V for 50 μs) using an Electro Cell Fusion Generator. After 2 h, the fused oocytes were selected, transferred to a 1-mm gab wire chamber overlaid with 10 μL 280 mM mannitol solution containing 0.1 mM MgSO4∙7H2O, 0.1 mM CaCl2∙2H2O, 0.5 mM HEPES, and 0.01% PVA, and then activated with 110 V DC for 50 μs using an Electro Cell Fusion Generator. The electro-stimulated oocytes were cultured in a chemically assisted activation medium supplemented with 1 μM oxamflatin for 4 h in an atmosphere with 5% CO_2_ at 38.5°C. Next, the activated embryos were transferred to IVC medium supplemented with 1 μM oxamflatin and further cultured for 20 h. After 20 h, the SCNT embryos were washed in IVC medium, and cultured in IVC medium in an atmosphere with 5% CO_2_ at 38.5°C. Blastocyst formation was evaluated on Day 6.

### TUNEL assay

To evaluate cell apoptotic blastomeres, a terminal deoxynucleotidyl transferase-mediated dUTP-digoxigenin nick end-labeling (TUNEL) assay was performed, as described previously [22]. Briefly, blastocysts were harvested from PA and SCNT on Day 6, washed three times in Dulbecco’s phosphate-buffered saline (DPBS)-PVA, fixed in 4% paraformaldehyde overnight at 4°C, and then permeabilized with 0.5% (v/v) Triton X-100 at room temperature for 1 h. Next, the blastocysts were blocked with 10 mg/mL BSA for 1 h, washed three times with DPBS-PVA, and stained with fluorescein-conjugated dUTP and terminal deoxynucleotidyl transferase for 1 h at 37°C. Subsequently, the blastocysts were washed three times with DPBS-PVA, and mounted on clean glass slides with DAPI staining. Then, the DAPI-labeled or TUNEL-positive nuclei were observed under a fluorescence microscope (Olympus). The numbers of total and apoptotic cells per blastocyst were determined by counting the nuclei with blue (DAPI) and green (TUNEL) signals; approximately more than five blastocysts per treatment group were used for the TUNEL assays in each independent experiment.

### Quantitative real-time polymerase chain reaction (qRT-PCR)

Extraction of poly(A) mRNA and cDNA synthesis were conducted as previously described [29]. Briefly, mRNA samples were extracted from 10 blastocysts using the Dynabeads mRNA Direct kit (Invitrogen Dynal AS, Oslo, Norway) according to the manufacturer’s instructions. Next, 300 μL of lysis/binding buffer was used for lysis of the blastocysts, 10 μL of Dynabeads oligo(dT)25 was added to separate the mRNAs, and the beads were hybridized for 5 min and separated from the binding buffer using a Dynal magnetic bar (Invitrogen). The beads with poly(A) mRNAs were washed using buffers A and B. After washing, 10 μL of Tris buffer was added to each tube for separation and the resulting poly(A) mRNAs were reverse-transcribed in a total volume of 20 μL with reactions containing oligo(dT)20, 5× RT buffer containing 25 mM Mg2+, 10 U of the RNase inhibitor ReverTra Ace (Toyobo, Osaka, Japan), and a 10 mM mixture of dNTPs. The secondary RNA structure was denatured by incubating the solution at 42°C for 60 min to facilitate cDNA production. The reaction was terminated by incubation at 99°C for 5 min and the synthesized cDNA was used as a template for polymerase chain reaction (PCR) amplification. The PCR conditions were as follows: 95°C for 30 s, 60°C for 30 s, and 72°C for 30 s, followed by extension at 72°C for 5 min. Quantitative real-time PCR (qRT-PCR) was performed using a Mx3000P QPCR system (Agilent, Santa Clara, CA, USA) with SYBR premix Ex Taq (Takara Bio Inc., Shiga, Japan) and the threshold cycle (Ct) was defined as the fractional cycle number at which the fluorescence passed a fixed threshold above baseline. For the comparative analyses, glyceraldehyde-3-phosphate dehydrogenase (GAPDH) was used for normalization, gene expression was expressed in terms of the fold change, and the 2−(SΔCT−CΔCT) method was used to analyze gene expression. The primers used in the present study are listed in S1 Table.

### Western blot analysis

The Western blot procedure was carried out as described previously [30]. Briefly, 20 oocytes from each group were washed twice with DPBS-PVA, lysed in 20 μL of lysis buffer (20 mM HEPES, 150 mM NaCl, 2 mM EGTA, 1 mM EDTA, 20 mM glycerol phosphate, 1% Triton X-100, and 10% glycerol) containing protease inhibitors for 2 h, and then boiled for 5 min at 100°C. The proteins were separated by 10% SDS-PAGE, transferred to nitrocellulose membranes (Millipore), and detected using immunoblotting. After blocking the membranes with 5% BSA in Tris pH 7.4, 150 mM NaCl, and 0.02% Tween-20 (TBST) overnight at 4°C, the membranes were blotted with primary antibodies against p-AKT (1:1,000, #4060L; Cell Signaling Technology), AKT (1:1,000, #9272S; Cell Signaling Technology), p-ERK (1:2,000, #4370L; Cell Signaling Technology), and ERK (1:2,000, #9102S; Cell Signaling Technology) overnight at 4°C. The blots were washed three times with TBST and then incubated with horseradish peroxidase-conjugated secondary antibodies. For the Western blot analysis, bands were visualized using an enhanced chemiluminescence detection reagent (ELPIS Biotech, Daejeon, South Korea) according to the manufacturer’s instructions.

### Statistical analysis

All experiments were repeated at least three times. Data are expressed as the means ± standard error of the mean (SEM). Data were compared using one-way analysis of variance, followed by Duncan’s multiple range test using SigmaStat software (SPSS Inc., Chicago, IL, USA). We used a *t*-test to analyze data from the U0126 treatment group. P-values less than 0.05 were considered statistically significant.

## Results

### Comparison of the developmental competence of class I and class II COCs

To examine the maturation competence and subsequent embryonic development according to COC morphology, PA-derived mature oocytes from class I and class II COCs were cultured in IVC medium for 6 days, and developmental parameters were scored on Day 6. To determine the precise molecular mechanisms underlying meiosis and oocyte competence in class I and II COCs, the COC cultures were performed in the absence of any growth factors. The development rate of class II COCs was significantly lower than that of class I COCs (Fig 1B and S2 Table). TUNEL staining analyses confirmed that fewer blastocysts were derived from class II COCs (Fig 1C and S3 Table), and that this group had a lower cell survival rate (Fig 1D and 1E and S3 Table). Consistent with this result, quantitative PCR analysis showed that gene expression of a pro-apoptotic gene (Bax) was higher in class II than in class I COCs, although they had slightly lower levels of another anti-apoptotic gene (Bcl-XL) compared to class I (Fig 1F). However, there was no significant between-group difference in nuclear maturation (Fig 1G and S4 Table).

### AKT signaling inhibition during early IVM induced developmental defects in pig embryos

**Fig 2:**
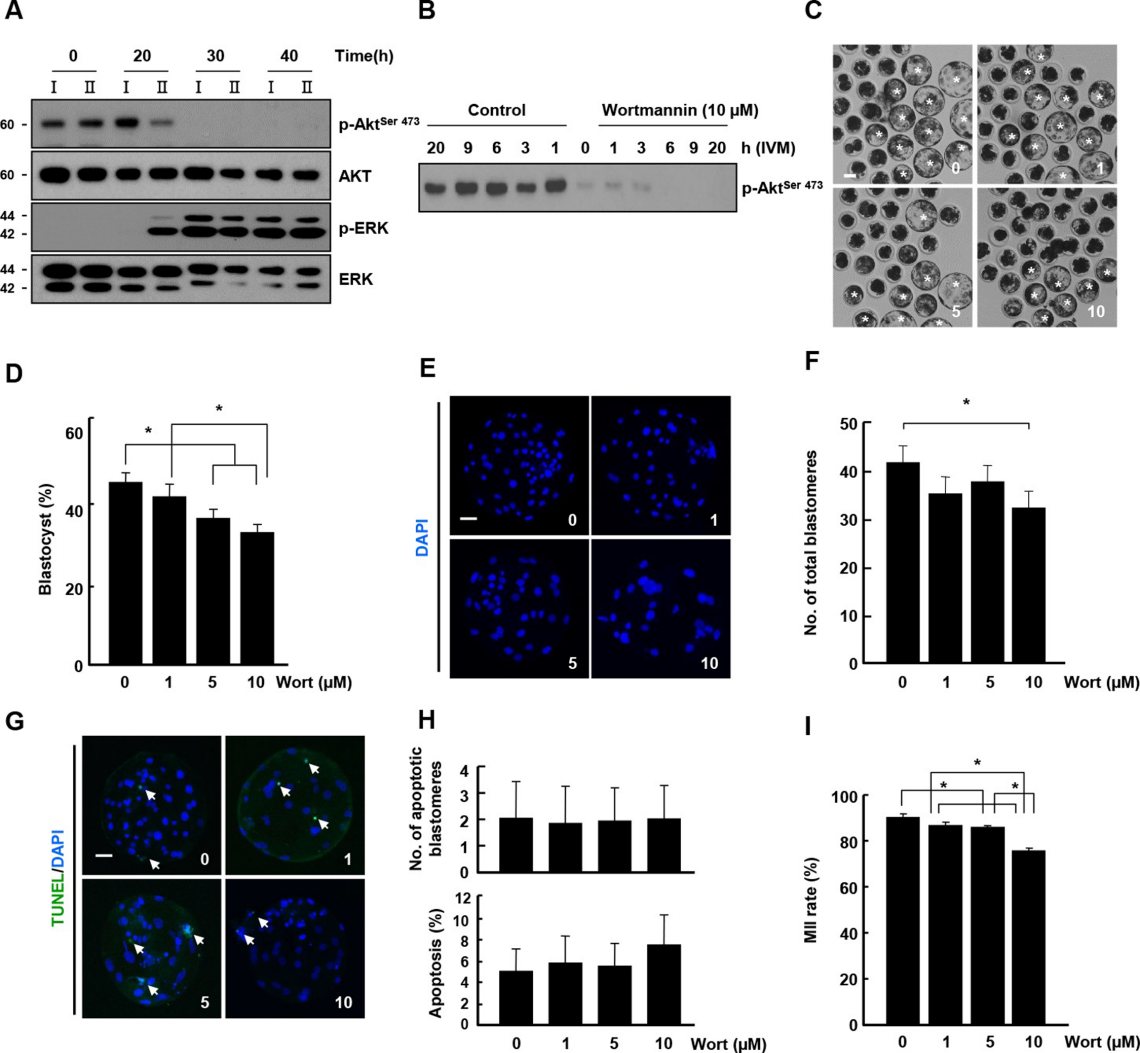
Inhibition of AKT signaling during early-IVM-induced developmental defects in class I COCs. (A) Western blot analysis (pAKT, AKT, pERK, and ERK) in oocytes from class I and class II COCs at each indicated time. (B) Effect of wortmannin (Wort) treatment on p-AKT expression at the indicated time. (C) Representative photographs of developed blastocysts at the indicated doses of wortmannin. Bar = 50 μm. (D) Blastocyst development rate in the indicated groups. The data are from three independent experiments, and the values represent the means ± SEM (*P < 0.05). (E) Nuclear staining of blastomeres using blastocysts cultured at the indicated doses of wortmannin. An image of DAPI (blue) signals is shown. Bar = 50 μm. (F) Quantification of the total number of cells in the indicated classes. The data are from three independent experiments, and the values represent the means ± SEM (*P < 0.05). (G) Apoptosis detection analysis in blastocysts at the indicated doses of wortmannin. Merged images (light green) between TUNEL (green, white arrow) and DAPI (blue) signals are shown. Bar = 50 μm. (H) Quantification of the number (upper) and proportion (lower) of apoptotic cells in the indicated classes. (I) Nuclear maturation rate of mature oocytes from the indicated classes. The data are from three independent experiments, and the values represent the means ± SEM (*P < 0.05).

To investigate the differences in kinase expression between class I and II COCs, western blot analysis was conducted using oocytes at each time point during IVM. The levels of p-AKT was lower in class II than in class I COCs at 20 h after IVM, whereas p-ERK levels was enhanced at the same time (Fig 2A). To examine the effects of wortmannin-mediated PI3K/AKT signaling during early IVM, class I COCs were cultured in early IVM media containing wortmannin with previously reported concentration (10 μM) and then further cultured in late IVM medium without inhibitor. Wortmannin decreased the immunoreactivity for p-AKT in a concentration-dependent manner (Fig 2B), and the blastocyst formation rate decreased in a dose-dependent manner (Fig 2C and 2D and S5 Table). Consistent with this observation, a 10 μM dose of wortmannin significantly decreased the total number of cells (Fig 2E and 2F and S6 Table). However, there was no significant difference in the number of apoptotic cells among the groups (Fig 2G and 2H and S6 Table). Meanwhile, wortmannin treatment delayed the nuclear development of class I COCs in a concentration-dependent manner (Fig 2I and S7 Table).

### EGF treatment improved porcine oocyte maturation via activation of AKT signaling

**Fig 3:**
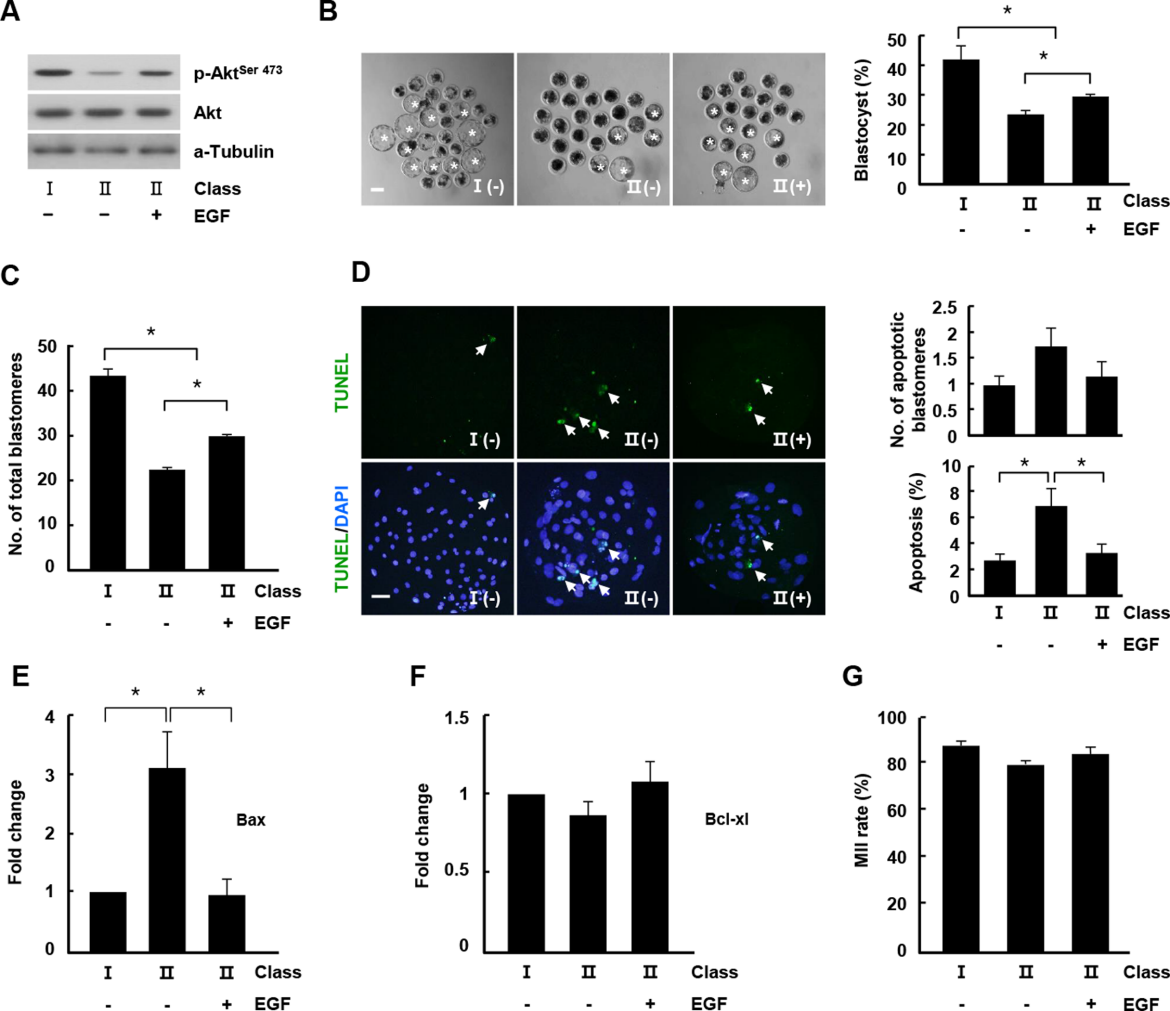
Maturation of porcine oocytes by EGF treatment via activation of AKT signaling. (A) Western blot analysis (p-AKT, AKT, and a-tubulin) was performed using oocytes from class I and class II COCs for each indicated group. (B) Effect of EGF treatment on the blastocyst formation rate of the indicated classes. Representative photographs of blastocysts (left) and blastocyst development rate of the indicated classes (right). Bar = 50 μm. The data are from three independent experiments, and the values represent the means ± SEM (*P < 0.05). (C) Quantification of the total number of cells in the indicated classes. The data are from three independent experiments, and the values represent the means ± SEM (*P < 0.05). (D) Apoptosis detection analysis of blastocysts from the indicated groups. Merged images (lower, light green, white arrow) between TUNEL (upper, green, and white arrow) and DAPI (blue) signals are shown. Bar = 50 μm. Quantification of the number (right, upper) and proportion (right, lower) of apoptotic cells in the indicated classes. (E and F) Relative mRNA expression levels of Bax and Bcl-XL in blastocysts from the indicated classes. The data are from three independent experiments, and the values represent the means ± SEM (*P < 0.05). (G) Nuclear maturation rate of mature oocytes from the indicated classes.

To investigate the effects of EGF-mediated PI3K/AKT signaling on porcine oocyte maturation, class II COCs were cultured in the presence of 10 ng/mL EGF during early IVM. EGF treatment increased the p-AKT band intensity, indicative of the activation of AKT signaling by EGF (Fig 3A). Mature oocytes were created via PA, and their developmental parameters were evaluated. EGF treatment increased the development rate to the blastocyst stage and the total number of blastocyst cells (Fig 3B and 3C and S8 and S9 Tables). TUNEL staining analyses revealed that the rate of apoptotic cells significantly decreased compared to the group without EGF (Fig 3D and S9 Table). Additionally, although the anti-apoptotic gene transcripts did not differ significantly among the groups, the pro-apoptotic transcripts were decreased in EGF-treated class II COCs (Fig 3E and 3F). Nuclear maturation was not affected by EGF treatment (Fig 3G and S10 Table). In contrast to the effects of wortmannin treatment on apoptosis and nuclear maturation, EGF-mediated AKT activation resulted in a reduction in cell death and did not affect nuclear maturation in class II COCs, indicating that AKT might have a different role in oocytes according to COC morphology.

### Transient inhibition of MAPK signaling increased both AKT activity and the capacity for blastocysts in PA and SCNT embryos

**Fig 4:**
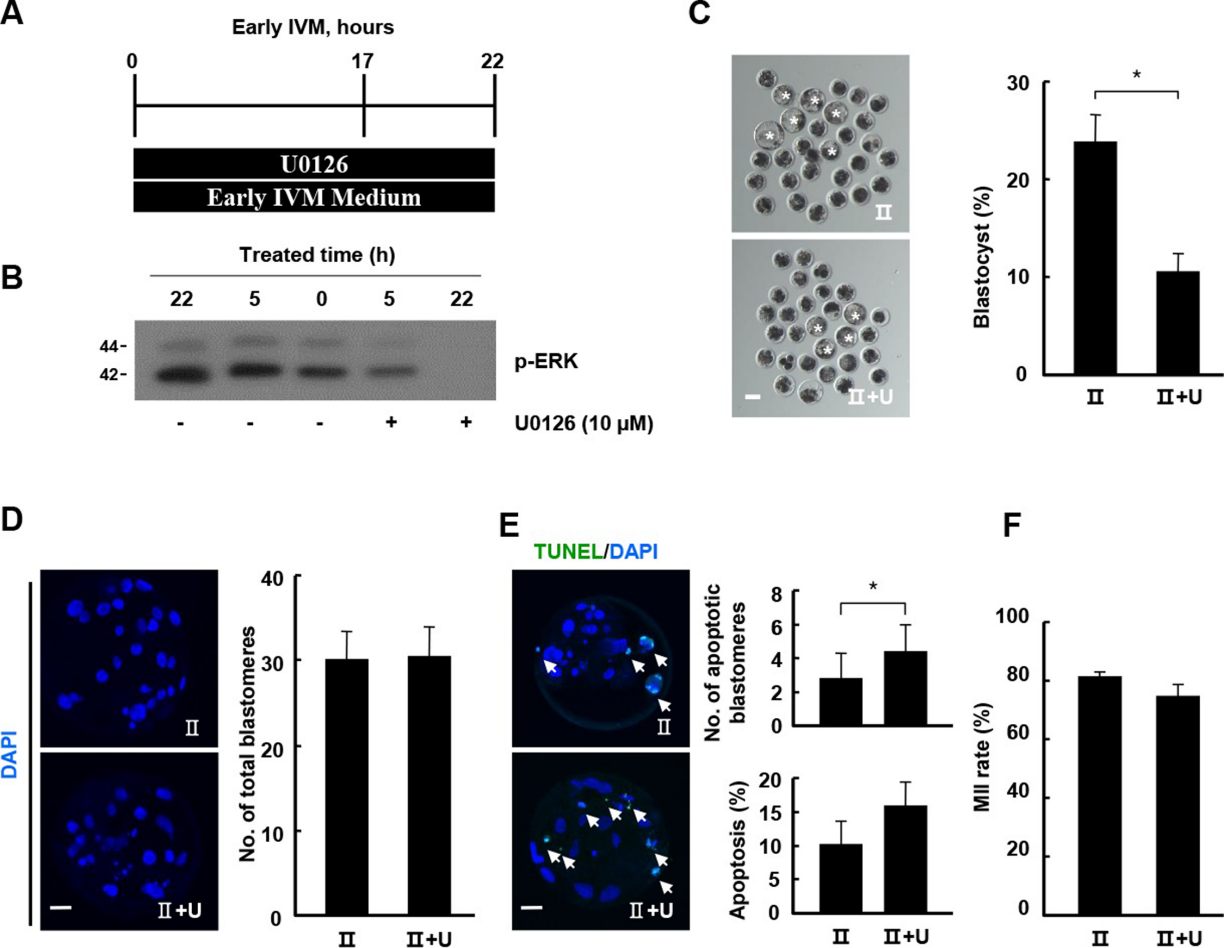
Effects of U0126 treatment during early IVM on the early development of porcine embryos. (A) Timeline of U0126 treatment during early IVM (0–22 h). (B) Western blot analysis (p-ERK) using oocytes derived from class II COCs cultured under the indicated treatment conditions. (C) Effect of U0126 treatment on blastocyst formation rates under the indicated treatment condition. Representative photographs of blastocysts (left) and blastocyst development rate under the indicated treatment conditions (right). Bar = 50 μm. The data are from three independent experiments, and the values represent the means ± SEM (*P < 0.05). (D) Nuclear staining of blastomeres using blastocysts cultured under the indicated treatment condition of U0126. An image of DAPI (blue) signals is shown. Bar = 50 μm. Quantification of the total number of cells under the indicated treatment condition of U0126 (right). (E) Apoptosis detection analysis of blastocysts under the indicated treatment conditions. Merged images (left, light green, white arrow) between TUNEL (green, white arrow) and DAPI (blue) signals are shown. Bar = 50 μm. Quantification of the number (right, upper) and proportion (right, lower) of apoptotic cells under the indicated treatment conditions. The data are from three independent experiments, and the values represent the means ± SEM (*P < 0.05). (F) Nuclear maturation rate of mature oocytes from the indicated classes.

**Fig 5:**
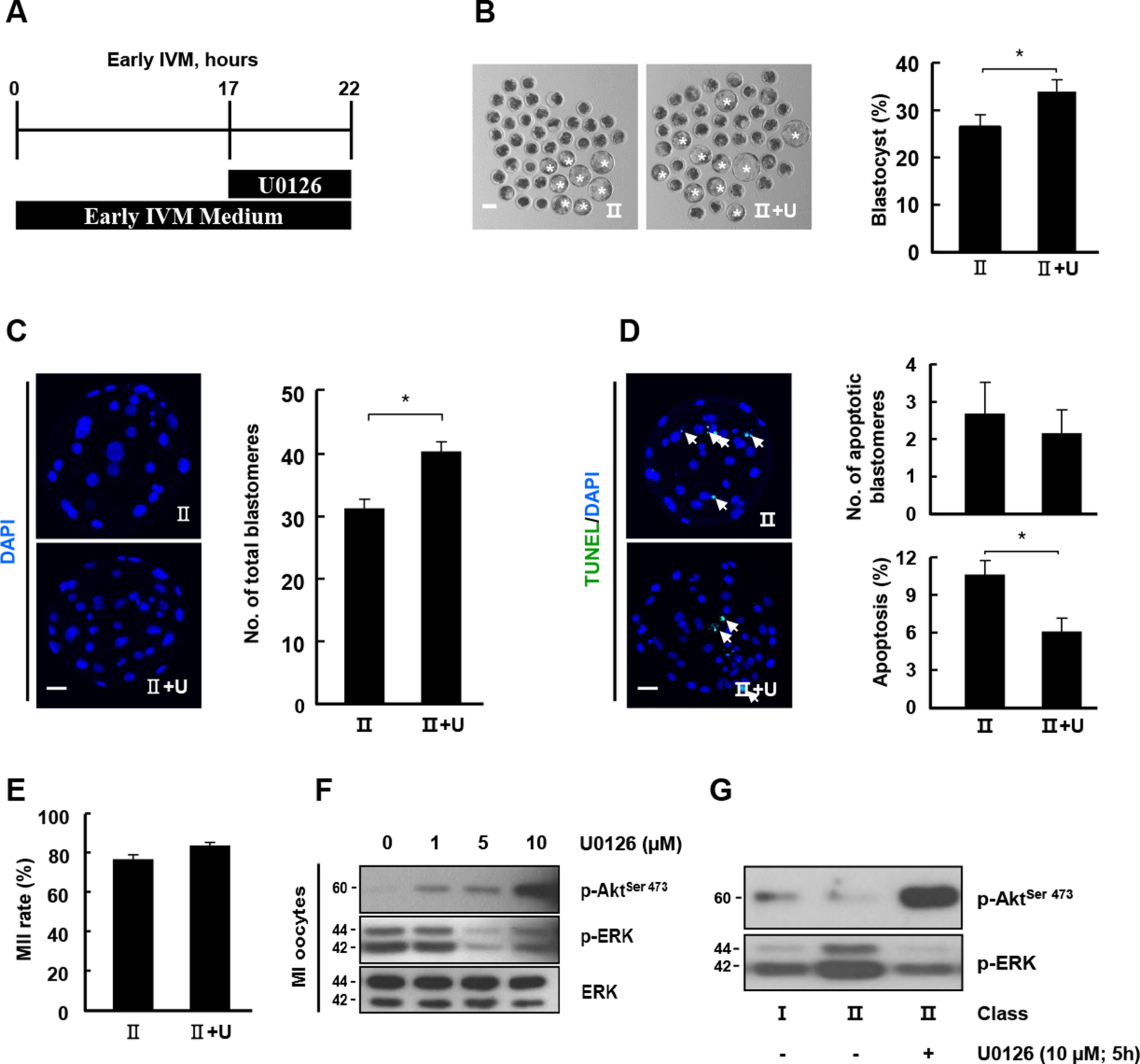
Transient inhibition of MAPK signaling increased both AKT activity and the capacity for blastocyst formation. (A) Timeline of U0126 treatment during early IVM (0–22 h). (B) Effect of U0126 treatment on the blastocyst formation rate under the indicated treatment condition. Representative photographs of blastocysts (left) and blastocyst development rate under the indicated treatment conditions (right). Bar = 50 μm. The data are from three independent experiments, and the values represent the means ± SEM (*P < 0.05). (C) Nuclear staining of blastomeres using blastocysts cultured under the indicated treatment condition of U0126. An image of DAPI (blue) signals is shown. Bar = 50 μm. Quantification of the total number of cells under the indicated treatment condition of U0126 (right). The data are from three independent experiments, and the values represent the means ± SEM (*P < 0.05). (D) Apoptosis detection analysis in blastocysts from the indicated treatment conditions. Merged images (left, light green, and white arrow) between TUNEL (green, white arrow) and DAPI (blue) signals are shown. Bar = 50 μm. Quantification of the number (right, upper) and proportion (right, lower) of apoptotic cells under the indicated treatment conditions. The data are from three independent experiments, and the values represent the means ± SEM (*P < 0.05). (E) Nuclear maturation rate of matured oocytes under the indicated treatment conditions. (F) Effects of U0126 treatment at the indicated doses on p-AKT levels. (G) Western blot of p-AKT and p-ERK using oocytes cultured under the indicated treatment conditions.

As p-ERK levels were increased in class II COCs compared with class I COCs after 20 h during IVM, class II COCs were cultured in early IVM media containing U0126 with previously reported concentration (10 μM) (Fig 4A). p-ERK levels were decreased after inhibitor treatment in a time-dependent manner (Fig 4B). Moreover, inhibitor treatment markedly decreased the blastocyst formation rate (Fig 4C and S11 Table) and total cell numbers in class II COCs (Fig 4D and S12 Table). U0126 treatment significantly increased the number of TUNEL-positive blastomeres compared with the control (Fig 4E and S12 Table). However, the effects of U0126 on nuclear maturation did not differ significantly between the two groups (Fig 4F and S13 Table). In a previous report, ERK activation occurred around 18 h during meiotic maturation [22]. To investigate the effect of transient inhibition on the MAPK signaling pathway, class II COCs were transferred to early IVM medium containing 10 μM U0126 during 17–22 h of IVM (Fig 5A); they were then further cultured in late IVM medium without U0126. The development rate was significantly higher than that of the control (Fig 5B and S14 Table) and the transient treatment group had more cells in total (Fig 5C and S15 Table) and higher survival rates than the control (Fig 5D and S15 Table). However, the number of mature oocytes in the transient treatment group was similar to that in the control group (Fig 5E and S16 Table). To investigate the regulation of the kinase signaling pathway according to differences in developmental parameters due to U0126, class II COCs were cultured at various U0126 concentrations for 22 h and subjected to western blotting. U0126 treatment increased the p-AKT band intensity in a concentration-dependent manner but decreased the p-ERK band intensity (Fig 5F). Consistent with this observation, p-AKT levels were enhanced in transiently inhibited oocytes compared with in the control (Fig 5G), which is indicative of downregulation of AKT signaling by MAPK signaling.

**Fig 6:**
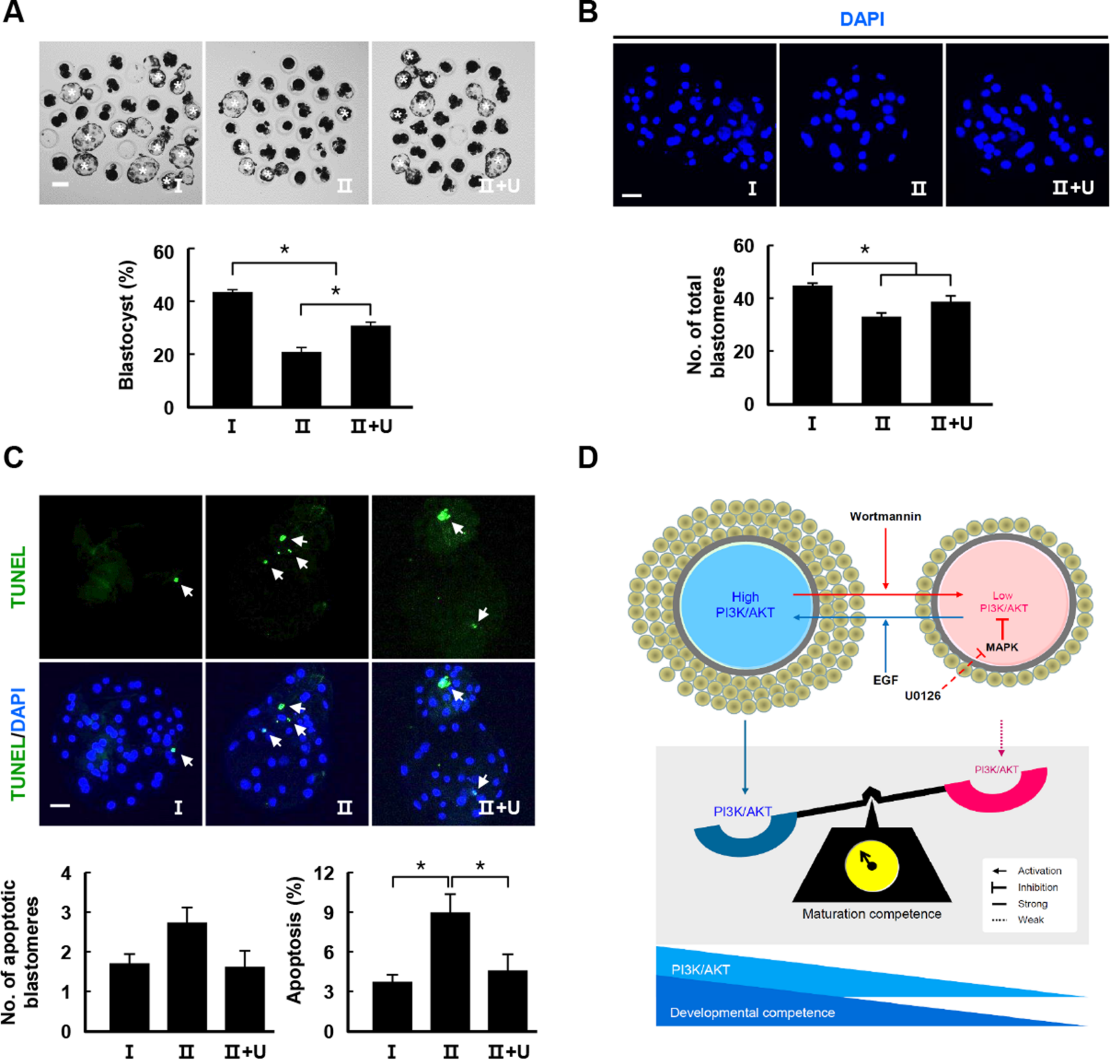
Effects of transient U0126 treatment during the early IVM phase on the developmental competence of SCNT embryos and a hypothetical model of the effects of kinase modulation on maturation competence. (A) Effects of U0126 treatment on the blastocyst formation rate under the transient inhibition condition. Representative photographs of blastocysts (upper) and the blastocyst development rate (lower) under the transient U0126 treatment condition. Bar = 50 μm. Data are from three independent experiments and the values represent means ± SEM (*P < 0.05). (B) Nuclear staining of blastomeres using blastocysts cultured under the transient U0126 treatment condition. Image showing DAPI (blue) signals (upper). Bar = 50 μm. Quantification of the total number of cells under the transient U0126 treatment condition (lower). Data are from three independent experiments and the values represent means ± SEM (*P < 0.05). (C) Apoptosis detection in blastocysts under the transient U0126 treatment condition. Merged images (mid green, light green, and white arrows) of TUNEL (green and white arrows) and DAPI (blue) signals are shown. Bar = 50 μm. Quantification of the number (bottom, left) and proportion (bottom, right) of apoptotic cells under the indicated treatment conditions. Data are from three independent experiments and the values represent means ± SEM (*P < 0.05). (D) The morphologically different COCs showed varying kinase expression levels during early IVM. Regulation of the PI3K/AKT pathway by wortmannin and EGF treatment determined maturation competence in class I and class II COCs, respectively. Moreover, increased activity in the PI3K/AKT signaling pathway via reduced MAPK activity led to improvements in maturation competence.

Next, we investigated whether modulation of the ERK pathway in class II COCs following treatment with U0126 would increase the early embryonic development of SCNT embryos. To investigate the effects of transient inhibition during early IVM, SCNT embryos derived from classified oocytes were cultured in IVC medium for 6 days either with or without transient ERK inhibition. Similar to the PA embryo results (Fig 5), transient U0126 treatment significantly increased the rates of blastocyst formation in SCNT embryos (Fig 6A and S17 Table). Moreover, several developmental parameters, including total blastomeres and cellular survival, were also increased in transiently treated class II COCs (Fig 6B and 6C and S18 Table).

## Discussion

Transgenic pigs are required for biomedical and regenerative medicine research due to their anatomical and physiological similarities to humans. Because the efficiency rate of producing transgenic animals is very low, oocytes with high developmental competence are needed to produce transgenic pigs. Thus, additional studies will be necessary to achieve a better understanding of the molecular mechanisms that govern the meiotic process underlying the development of oocytes, to yield more efficient methods of oocyte maturation. The present study was the first to demonstrate the distinct activities of two signaling pathways, PI3K/AKT and MAPK, between class I and II COCs in the pig after 20 h during IVM. It was shown that modulation of these pathways resulted in lower developmental competence in class II COCs compared to class I COCs. In particular, activation of the PI3K/AKT pathway was critical for oocyte maturation, while the MAPK/ERK pathway negatively regulated meiotic progression by impeding the AKT cascade. Additionally, EGF was identified as a positive regulator of oocyte competence in class II COCs following AKT activation, which suggests that the regulation of signaling pathways such as MAPK/ERK and PI3K/AKT influences the behavior of porcine oocytes. Therefore, the present results will further current understanding of the molecular mechanisms underlying the meiotic process in the pig oocyte. To determine the developmental capacity of COCs, porcine COCs were classified according to the number of cumulus cell layers and were cultured in IVM medium. In a previous study, most COCs obtained from ovaries consisted of less than two layers of cumulus cells, and the proportion of COCs with five or more layers of cumulus cells was below 10% [2]. The present study identified distinct types of developmental competence and kinase activity between oocytes derived from class I and class II COCs, and also provided possible novel methods for increasing the competence of oocytes and early embryonic development via the regulation of signaling pathways.

The PI3K/AKT signaling pathway plays a critical role in growth factor-promoted cell proliferation. AKT is activated following the induction of phosphorylation at two key residues, Thr308 and Ser473, and exerts distinct cellular effects based on these phosphorylation sites. Similarly, Thr308 and Ser473 are regulated by specific kinases: phosphoinositide-dependent kinase 1 and rapamycin complex 2, respectively [23, 24]. Phosphorylation at Thr308 is associated with anti-apoptotic functions, whereas phosphorylation at Ser473 regulates cell survival and proliferation [25]. Activated AKT is involved in the resumption of meiosis in mammalian oocytes [9, 10, 26] and phosphorylation at either Ser473 or Thr308 has been detected at different stages of oocyte maturation. For example, phospho-Ser473 is detected around GVBD after 15 h of culture and phospho-Thr308 is detected at 24 h intervals in the culture [10]. Additionally, phospho-Ser473 is localized on the nuclear membrane and the centrosomal area, while phospho-Thr308 is only found on the centrosome, which indicates distinct localization based on the phosphorylation site [27]. The present immunoblotting analyses revealed that AKT was present in both the nuclei and cytoplasm of oocytes, while there was greater accumulation of phospho-AKT in the nuclear region of class I COCs compared to class II COCs. However, there were higher levels of phospho-ERK in the cytoplasm of class II COCs compared to class I COCs, even though considerable ERK phosphorylation was detected in the nuclei of class II COCs. The present study was the first to identify different subcellular localizations of signaling molecules based on the classification of COCs, which indicates that distinct targets were involved in the oocyte maturations of class I versus II COCs (S1 Fig). Regarding the completion of meiosis, AKT phosphorylated at Ser473 is associated with the emission of a second polar body, whereas AKT phosphorylated at Thr308 affects the organization of microtubules. In particular, AKT phosphorylated at phospho-Ser473 activates Forkhead box O3a (FOXO3a) via the insulin signaling pathway, to subsequently regulate functional ovarian follicles [28, 29]. Therefore, it is possible that the activation of AKT by phosphorylation at Ser473 enhanced developmental competency via FOXO3a; however, this will require further study.

In particular, we observed that the expression of phosphorylated Ser473 at 20 h during IVM was lower in class II COCs than in class I COCs. Thus, we confirmed that inhibition of AKT phosphorylation during early IVM decreased the developmental competence of class I COCs. In previous studies, the interference of the PI3K/AKT signaling pathway impeded meiotic events, such as the MI–MII transition, spindle organization, polar body extrusion, and microtubule organization [10, 26]. Another study showed that ganglioside treatment suppressed the meiotic process via EGFR-mediated PI3K/AKT signaling in pigs [14]. Consistent with this result, we showed that suppression of AKT phosphorylation at Ser473 greatly decreased developmental competence. By contrast, growth factor-mediated AKT activation improved somatic cell proliferation and the developmental capacity of embryos. COCs are typically cultured in an EGF-containing medium to assess the early developmental competence of oocyte maturation [30, 31] whereas they are maintained in an EGF-depleted medium to assess the induction of meiosis [10, 11]. The present study showed that the PI3K/AKT pathway regulated oocyte maturation and increased cell survivability and, for the first time, demonstrated that EGF enhances oocyte maturation by upregulating AKT activity. These results indicate that the difference in AKT activity is one of the causes of the difference in developmental competence between class I and class II COCs.

The PI3K/Akt signaling pathway has a critical role in mediating survival signals depending on cell type and context [32, 33]. In mammalian COCs, the high activity of PI3-kinase protects against induction of apoptosis during IVM [34]. AKT activity in cumulus cells is required for resistance to cell death and spontaneous meiotic resumption [34]. In this study, there were more apoptotic cells and pro-apoptotic transcripts in blastocysts derived from class II COCs than in those from class I COCs. Although wortmannin had no effects on apoptosis induction in class I COCs, EGF-mediated AKT activation significantly decreased the number of apoptotic cells in blastocysts derived from class II COCs. Therefore, we suggest that AKT has a role in protecting oocytes from DNA damage in class II COCs, and the role of AKT might differ between class I and class II COCs.

ERK1/2 is a Ser/Thr kinase that is activated by MEK-dependent phosphorylation upon stimulation of cells by various growth factors. The main effects of ERK1/2 include S phase suppression, MII arrest maintenance, mitotic microtubule organization, and maintenance of meiotic spindle morphology [35, 36]. ERK1/2 activation occurs at different stages according to species, and the oocytes of the pig, cow, and *Xenopus* are activated before GVBD [18, 37, 38], whereas p-ERK1/2 is expressed during the MII stage. In this study, phosphorylated ERK1/2 levels were higher in oocytes derived from class II COCs than from those derived from class I COCs at 20 h during IVM. Thus, we confirmed that inhibition of the ERK1/2 signaling pathway by U0126 from 0 to 22 h of IVM resulted in a reduction in both the blastocyst formation rate and the cell survival rate. Consistent with these results, in the mouse, ERK1/2 deletion resulted in developmental defects associated with the maternal–zygotic transition [19]. Additionally, the inhibitory effect of U0126 led to the suppression of MI–MII transition and subsequent events [39, 40]. However, Zhang et al. [41] found that U0126 treatment arrest had no effect on nuclear maturation from 24 to 44 h of IVM and determined that activation of ERK1/2 was essential early in the resumption of meiosis. Consistent with the above reports, class II COCs were cultured in early IVM medium with U0126, which inhibited cytoplasmic maturation but not nuclear maturation. The effects of U0126 on meiotic maturation mostly blocked or delayed the nuclear process in oocytes. However, our results showed that transient treatment of U0126 at 17–22 h of IVM improved developmental competence in class II COCs. Previous studies have attempted to enhance maturation competence (e.g., cytoplasmic maturation) using meiotic inhibitors involving hypoxanthine [42] and cAMP analogs [43], which led to the synchronization of meiosis in immature oocytes. Additionally, Zhang et al. [41] suggested that the ERK cascade regulated the degradation of maternal transcripts during meiotic progression. Therefore, our results suggest that the effects of transient inhibition by U0126 may greatly affect meiotic synchronization and the maintenance of maternal factors in oocytes.

AKT activity has a key role in the MI–MII transition during meiotic progression, but it is not essential for inducing GVBD [10]. Additionally, ERK signaling suppressed PI3K/AKT signaling [44] and upregulated growth factor-mediated AKT activation via MEK inhibition [45]. In the present study, we showed that transient inhibition of MAPK enhanced developmental competence in class II COCs. Thus, we investigated the regulatory mechanism underlying U0126-mediated ERK and AKT expression in oocytes. We confirmed that AKT activity was increased by U0126 in a concentration-dependent manner and enhanced by transient inhibition in class II COCs according to a decrease in ERK phosphorylation (Fig 5). This suggests that transient inhibition of MAPK may enhance cytoplasmic maturation of class II COCs via activation of the PI3K/AKT signaling pathway. Therefore, we suggest that AKT activity during early IVM may have a critical role in meiotic progression (Fig 6D).

An increasing number of reports have demonstrated the critical role that mRNA translation plays in protein synthesis during the progression of meiosis. It is well-established that mRNA translation is mediated by the rapamycin (mTOR) pathway via the PI3K/AKT pathway in somatic cells [46]. The activated mTOR complex 1 (mTORC1) stimulates the S6 ribosoma protein and eukaryotic initiation factor 4E to increase the translations of VEGF, FGF-2, and cyclin D1 [47, 48]. It has also been reported in mice that the PI3K-AKT-mTOR signaling cascade influences the translation of maternal mRNA and early embryonic developmental competence during meiosis by regulating cumulus cells [49]. The present study demonstrated distinct activation of AKT based on COC class and showed that these differences might affect the translation of maternal mRNA and subsequent oocyte competence. The activation of AKT by EGF treatment, and the enhancement of developmental competence in class II COCs, may constitute evidence that the AKT/mTOR pathway induced oocyte maturation by regulating the translation of maternal mRNA, as has been shown in the mouse system; however, further studies will be required.

In conclusion, the present study demonstrated that the activity of two kinases differed between class I and class II COCs during meiotic progression. The regulation of AKT activity through use of EGF for early IVM altered the blastocyst formation rate, number of cells, and survival rate and appeared to be associated with cytoplasmic maturation. Moreover, transient inhibition of ERK1/2 in class II COCs increased developmental competence by enhancing AKT activity, possibly related to negative regulation. These results may provide a strategy for increasing the utilization rate of COCs and further understanding meiotic processes.

## Supporting information

S1 Fig Subcellular localizations of AKT and ERK: comparison between class I and class II oocytes. Western blotting of AKT (A) and ERK (B) using oocytes matured in the indicated groups at 20 h during IVM. The data are from three independent experiments, and values represent the means ± SE (*P < 0.05).
(TIF)

S1 Table Primer sequences used for qRT-PCR. (PDF)

S2 Table Development of matured porcine oocytes derived from different types of COCs. (PDF)

S3 Table Cell numbers and cellular survival rates of porcine PA blastocysts derived from different types of COCs. (PDF)

S4 Table Nuclear maturation derived from different types of cumulus-oocyte complexes (COCs). (PDF)

S5 Table Effects of wortmannin treatment during the early IVM phase on developmental competence. (PDF)

S6 Table Effects of wortmannin treatment during the early IVM phase on cell number and cellular survival in porcine PA blastocysts. (PDF)

S7 Table Effects of wortmannin treatment during the early IVM phase on nuclear maturation. (PDF)

S8 Table Effects of EGF treatment during the early IVM phase on developmental competence. (PDF)

S9 Table Effects of EGF treatment during the early IVM phase on cell number and cellular survival in porcine PA blastocysts. (PDF)

S10 Table Effects of EGF treatment on in vitro porcine oocyte maturation. (PDF)

S11 Table Effects of U0126 treatment during the early IVM phase on developmental competence. (PDF)

S12 Table Effects of U0126 treatment during the early IVM phase on cell number and cellular survival in porcine PA blastocysts. (PDF)

S13 Table Effects of U0126 treatment during the early IVM phase on in vitro porcine oocyte maturation. (PDF)

S14 Table Effects of transient U0126 treatment during the early IVM phase on developmental competence. (PDF)

S15 Table Effects of transient U0126 treatment during the early IVM phase on cell number and cellular survival of porcine PA blastocysts. (PDF)

S16 Table Effects of transient U0126 treatment during the early IVM phase on in vitro porcine oocyte maturation. (PDF)

S17 Table Effects of transient U0126 treatment during the early IVM phase on the developmental competence of SCNT embryos. (PDF)

S18 Table Effects of transient U0126 treatment during the early IVM phase on cell number and cellular survival in porcine SCNT blastocysts. (PDF)
