# Supplementary figures and images for: The effects of kinase modulation on in vitro maturation according to different cumulus-oocyte complex morphologies
Source: PLoS One. 2018 Oct 11;13(10):e0205495. doi: 10.1371/journal.pone.0205495 (PMC6181369; doi:10.1371/journal.pone.0205495)

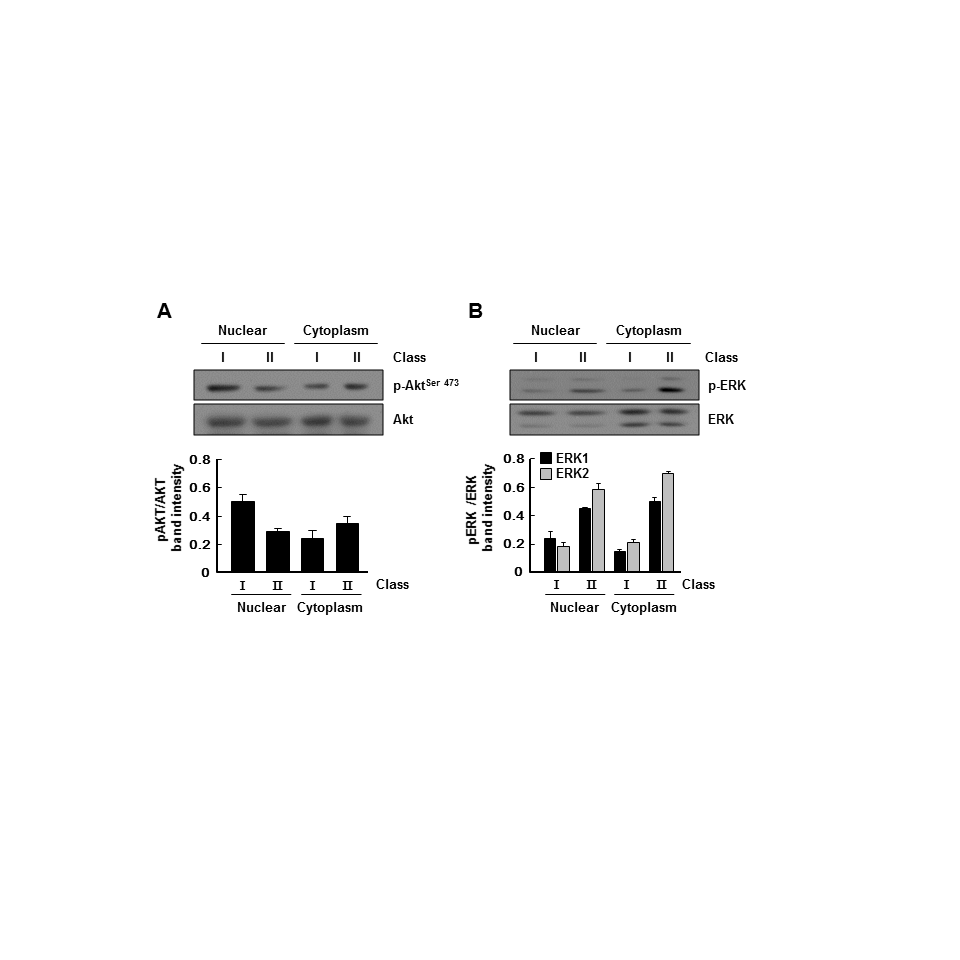

Supplement: S1 Fig — Western blotting of AKT (A) and ERK (B) using oocytes matured in the indicated groups at 20 h during IVM. The data are from three independent experiments, and values represent the means ± SE (*P < 0.05). (TIF) [file pone.0205495.s001.tif]
