# Supplementary material for: The effects of kinase modulation on in vitro maturation according to different cumulus-oocyte complex morphologies
Source: PLoS One. 2018 Oct 11;13(10):e0205495. doi: 10.1371/journal.pone.0205495 (PMC6181369; doi:10.1371/journal.pone.0205495)
Supplement: S1 Table — (PDF) [file pone.0205495.s002.pdf]

Supplementary Table S1. Primer sequences used for qRT-PCR

Gene	Primer sequences	GenBank accession no.	Product size (bp)
Bax	F: 5'-CTA CTT TGC CAG TAA ACT GG -3'<br>R: 5'-TCC CAA AGT AGG AGA GGA -3'	XM_005664710.1	158
Bcl-xl	F: 5'-AGG GCA TTC AGT GAC CTG AC -3'<br>R: 5'-TGG ATC CAA GGC TCT AGG TG-3'	NM_214285.1	242
GAPDH	F: 5'-TCG GAG TGA ACG GAT TTG GC-3'<br>R: 5'-TGC CGT GGG TGG AAT CAT AC-3'	NM_001206359.1	147
