# Supplementary material for: The effects of kinase modulation on in vitro maturation according to different cumulus-oocyte complex morphologies
Source: PLoS One. 2018 Oct 11;13(10):e0205495. doi: 10.1371/journal.pone.0205495 (PMC6181369; doi:10.1371/journal.pone.0205495)
Supplement: S2 Table — (PDF) [file pone.0205495.s003.pdf]

Supplementary Table S2. Development of matured porcine oocytes derived from different types of COCs

Class	No. of embryos used	No. (%)* of blastocysts developed
I	100	44 (46.0 ± 0.3)[a]
II	136	41 (30.7 ± 4.2)[b]

Data are presented as means ± SEM. Values within a column with different superscript letters differ significantly ($p < 0.05$).

*Blastocyst development rate = (no. of blastocysts developed/no. of embryos used) × 100.
