# Supplementary material for: The effects of kinase modulation on in vitro maturation according to different cumulus-oocyte complex morphologies
Source: PLoS One. 2018 Oct 11;13(10):e0205495. doi: 10.1371/journal.pone.0205495 (PMC6181369; doi:10.1371/journal.pone.0205495)
Supplement: S3 Table — (PDF) [file pone.0205495.s004.pdf]

Supplementary Table S3. Cell numbers and cellular survival rates of porcine PA blastocysts derived from different types of COCs

Class	No. of blastocysts used	No. of blastomeres	No. of apoptotic cells (%)*
I	19	57.9 ± 2.5^{a}	1.7 ± 0.1^{a} $(2.8 \pm 0.3)^{a}$
II	14	38.9 ± 1.9^{b}	2.4 ± 0.2^{b} $(6.7 \pm 1.5)^{b}$

Data are presented as means ± SEM. Values within a column with different superscript letters differ significantly ($p < 0.05$).

*Apoptosis rate = (no. of apoptotic cells/no. of total cells in blastocyst) × 100.
