# Supplementary material for: The effects of kinase modulation on in vitro maturation according to different cumulus-oocyte complex morphologies
Source: PLoS One. 2018 Oct 11;13(10):e0205495. doi: 10.1371/journal.pone.0205495 (PMC6181369; doi:10.1371/journal.pone.0205495)
Supplement: S4 Table — (PDF) [file pone.0205495.s005.pdf]

Supplementary Table S4. Nuclear maturation derived from different types of cumulus-oocyte complexes (COCs)

Class	No. of COCs	No. (%) of oocytes with PB
I	185	164 (88.3 ± 1.7)
II	278	236 (82.9 ± 2.4)

Data are presented as means ± SEM.
