# Supplementary material for: The effects of kinase modulation on in vitro maturation according to different cumulus-oocyte complex morphologies
Source: PLoS One. 2018 Oct 11;13(10):e0205495. doi: 10.1371/journal.pone.0205495 (PMC6181369; doi:10.1371/journal.pone.0205495)
Supplement: S5 Table — (PDF) [file pone.0205495.s006.pdf]

Supplementary Table S5. Effects of wortmannin treatment during the early IVM phase on developmental competence

Wortmannin (uM)	No. of oocytes used	No. (%)* of blastocysts developed
0	147	66 (46.5 ± 1.5)[a,c]
1	134	53 (42.3 ± 2.4)[a,c]
5	137	51 (34.4 ± 1.2)[b,c]
10	130	43 (31.3 ± 1.3)[b,d]

Data are presented as means ± SEM. Values within a column with different superscript letters differ significantly ($p < 0.05$).

*Blastocyst development rate = (no. of blastocysts developed/no. of embryos used) × 100.
