# Supplementary material for: The effects of kinase modulation on in vitro maturation according to different cumulus-oocyte complex morphologies
Source: PLoS One. 2018 Oct 11;13(10):e0205495. doi: 10.1371/journal.pone.0205495 (PMC6181369; doi:10.1371/journal.pone.0205495)
Supplement: S6 Table — (PDF) [file pone.0205495.s007.pdf]

**Supplementary Table S6.** Effects of wortmannin treatment during the early IVM phase on cell number and cellular survival in porcine PA blastocysts

| Wortmannin (uM) | No. of blastocysts used | No. of blastomeres | No. of apoptotic cells (%)* |
|---|---|---|---|
| 0 | 27 | 41.6 ± 4.0[a] | 2.2 ± 1.5 (5.0 ± 2.0) |
| 1 | 51 | 36.2 ± 3.0[a] | 1.8 ± 1.3 (5.7 ± 2.8) |
| 5 | 50 | 37.7 ± 2.9[a] | 1.9 ± 1.1 (5.3 ± 2.1) |
| 10 | 36 | 31.5 ± 3.0[b] | 2.0 ± 1.4 (7.4 ± 2.9) |

Data are presented as means ± SEM. Values within a column with different superscript letters differ significantly ($p < 0.05$).

*Apoptosis rate = (no. of apoptotic cells/no. of total cells in blastocyst) × 100.
