# Supplementary material for: The effects of kinase modulation on in vitro maturation according to different cumulus-oocyte complex morphologies
Source: PLoS One. 2018 Oct 11;13(10):e0205495. doi: 10.1371/journal.pone.0205495 (PMC6181369; doi:10.1371/journal.pone.0205495)
Supplement: S7 Table — (PDF) [file pone.0205495.s008.pdf]

Supplementary Table S7. Effects of wortmannin treatment during the early IVM phase on nuclear maturation

Wortmannin (uM)	No. of COCs	No. (%) of oocytes with PB
0	162	147 (91.0 ± 1.5)^{a,b,c}
1	155	134 (86.9 ± 1.6)^{a,b,c}
5	156	137 (87.8 ± 0.9)^{b}
10	175	130 (74.3 ± 1.5)^{d}

Data are presented as means ± SEM. Values within a column with different superscript letters differ significantly ($p < 0.05$).
