# Supplementary material for: The effects of kinase modulation on in vitro maturation according to different cumulus-oocyte complex morphologies
Source: PLoS One. 2018 Oct 11;13(10):e0205495. doi: 10.1371/journal.pone.0205495 (PMC6181369; doi:10.1371/journal.pone.0205495)
Supplement: S8 Table — (PDF) [file pone.0205495.s009.pdf]

Supplementary Table S8. Effects of EGF treatment during the early IVM phase on developmental competence

Class	No. of embryos used	No. (%)* of blastocysts developed
I	139	59 (41.8 ± 4.7)[a]
II	214	51 (23.3 ± 1.5)[b]
II+EGF	243	72 (29.4 ± 0.8)[b]

Data are presented as means ± SEM. Values within a column with different superscript letters differ significantly ($p < 0.05$).

*Blastocyst development rate = (no. of blastocysts developed/no. of embryos used) × 100.
