# Supplementary material for: The effects of kinase modulation on in vitro maturation according to different cumulus-oocyte complex morphologies
Source: PLoS One. 2018 Oct 11;13(10):e0205495. doi: 10.1371/journal.pone.0205495 (PMC6181369; doi:10.1371/journal.pone.0205495)
Supplement: S9 Table — (PDF) [file pone.0205495.s010.pdf]

Supplementary Table S9. Effects of EGF treatment during the early IVM phase on cell number and cellular survival in porcine PA blastocysts

Class	No. of blastocysts used	No. of blastomeres	No. of apoptotic cells (%)*
I	21	40.3 ± 3.9[a]	1.0 ± 0.93 (2.6 ± 1.5)[a]
II	17	26.8 ± 3.13[b]	1.7 ± 1.2 (6.8 ± 2.4)[b]
II+EGF	15	32.7 ± 3.13[b]	1.2 ± 0.9 (3.5 ± 1.4)[a]

Data are presented as means ± SEM. Values within a column with different superscript letters differ significantly ($p < 0.05$).

*Apoptosis rate = (no. of apoptotic cells/no. of total cells in blastocyst) × 100.
