# Supplementary material for: The effects of kinase modulation on in vitro maturation according to different cumulus-oocyte complex morphologies
Source: PLoS One. 2018 Oct 11;13(10):e0205495. doi: 10.1371/journal.pone.0205495 (PMC6181369; doi:10.1371/journal.pone.0205495)
Supplement: S10 Table — (PDF) [file pone.0205495.s011.pdf]

Supplementary Table S10. Effects of EGF treatment on in vitro porcine oocyte maturation

Class	No. of COCs	No. (%) of oocytes with PB
I	145	128 (83.3 ± 1.9)
II	163	131 (80.1 ± 1.6)
II+EGF	202	172 (84.1 ± 2.3)

Data are presented as means ± SEM.
