# Supplementary material for: The effects of kinase modulation on in vitro maturation according to different cumulus-oocyte complex morphologies
Source: PLoS One. 2018 Oct 11;13(10):e0205495. doi: 10.1371/journal.pone.0205495 (PMC6181369; doi:10.1371/journal.pone.0205495)
Supplement: S11 Table — (PDF) [file pone.0205495.s012.pdf]

Supplementary Table S11. Effects of U0126 treatment during the early IVM phase on developmental competence

Class	No. of embryos used	No. (%)* of blastocysts developed
II	114	26 (21.7 ± 2.9)[a]
II+U0126	117	15 (12.3 ± 2.0)[b]

Data are presented as means ± SEM. Values within a column with different superscript letters differ significantly ($p < 0.05$).

*Blastocyst development rate = (no. of blastocysts developed/no. of embryos used) × 100.
