# Supplementary material for: The effects of kinase modulation on in vitro maturation according to different cumulus-oocyte complex morphologies
Source: PLoS One. 2018 Oct 11;13(10):e0205495. doi: 10.1371/journal.pone.0205495 (PMC6181369; doi:10.1371/journal.pone.0205495)
Supplement: S12 Table — (PDF) [file pone.0205495.s013.pdf]

Supplementary Table S12. Effects of U0126 treatment during the early IVM phase on cell number and cellular survival in porcine PA blastocysts

Class	No. of blastocysts used	No. of blastomeres	No. of apoptotic cells (%)[*]
II	20	30.1 ± 3.7	2.8 ± 1.5[a] (10.2 ± 3.1)
II+U0126	20	30.5 ± 3.5	4.4 ± 1.6[b] (15.9 ± 3.5)

Data are presented as means ± SEM. Values within a column with different superscript letters differ significantly ($p < 0.05$).

[*]Apoptosis rate = (no. of apoptotic cells/no. of total cells in blastocyst) × 100.
