# Supplementary material for: The effects of kinase modulation on in vitro maturation according to different cumulus-oocyte complex morphologies
Source: PLoS One. 2018 Oct 11;13(10):e0205495. doi: 10.1371/journal.pone.0205495 (PMC6181369; doi:10.1371/journal.pone.0205495)
Supplement: S15 Table — (PDF) [file pone.0205495.s016.pdf]

**Supplementary Table S15.** Effects of transient U0126 treatment during the early IVM phase on cell number and cellular survival of porcine PA blastocysts

| Class | No. of blastocysts used | No. of blastomeres | No. of apoptotic cells (%)* |
|---|---|---|---|
| II | 20 | 31.0 ± 1.4[a] | 2.7 ± 0.9 (9.0 ± 1.0)[a] |
| II+U0126 | 20 | 40.0 ± 1.6[b] | 2.1 ± 0.6 (5.1 ± 1.0)[b] |

Data are presented as means ± SEM. Values within a column with different superscript letters differ significantly ($p < 0.05$).

*Apoptosis rate = (no. of apoptotic cells/no. of total cells in blastocyst) × 100.
