# Supplementary material for: The effects of kinase modulation on in vitro maturation according to different cumulus-oocyte complex morphologies
Source: PLoS One. 2018 Oct 11;13(10):e0205495. doi: 10.1371/journal.pone.0205495 (PMC6181369; doi:10.1371/journal.pone.0205495)
Supplement: S16 Table — (PDF) [file pone.0205495.s017.pdf]

Supplementary Table S16. Effects of transient U0126 treatment during the early IVM phase on in vitro porcine oocyte maturation

Class	No. of COCs	No. (%) of oocytes with PB
II	134	110 (76.6 ± 2.0)
II+U0126	151	127 (83.6 ± 2.0)

Data are presented as means ± SEM.
