# Supplementary material for: The effects of kinase modulation on in vitro maturation according to different cumulus-oocyte complex morphologies
Source: PLoS One. 2018 Oct 11;13(10):e0205495. doi: 10.1371/journal.pone.0205495 (PMC6181369; doi:10.1371/journal.pone.0205495)
Supplement: S17 Table — (PDF) [file pone.0205495.s018.pdf]

Supplementary Table S17. Effects of transient U0126 treatment during the early IVM phase on the developmental competence of SCNT embryos

Class	No. of embryos used	No. (%)* of blastocysts developed
I	144	62 (43.1 ± 1.1)[a]
II	114	24 (20.5 ± 2.3)[b]
II+U0126	113	35 (30.5 ± 1.5)[c]

Data are presented as means ± SEM. Values within a column with different superscript letters differ significantly ($p < 0.05$).

*Blastocyst development rate = (no. of blastocysts developed/no. of embryos used) × 100.
