# Supplementary material for: The effects of kinase modulation on in vitro maturation according to different cumulus-oocyte complex morphologies
Source: PLoS One. 2018 Oct 11;13(10):e0205495. doi: 10.1371/journal.pone.0205495 (PMC6181369; doi:10.1371/journal.pone.0205495)
Supplement: S18 Table — (PDF) [file pone.0205495.s019.pdf]

Supplementary Table S18. Effects of transient U0126 treatment during the early IVM phase on cell number and cellular survival in porcine SCNT blastocysts

Class	No. of blastocysts used	No. of blastomeres	No. of apoptotic cells (%)*
I	40	44.5 ± 1.2^{a}	1.7 ± 0.3 $(3.7 \pm 0.6)^{a}$
II	17	32.8 ± 1.8^{b}	2.7 ± 0.4 $(9.0 \pm 1.4)^{b}$
II+U0126	18	38.6 ± 2.5^{b}	1.6 ± 0.4 $(4.6 \pm 1.2)^{a}$

Data are presented as means ± SEM. Values within a column with different superscript letters differ significantly ($p < 0.05$).

*Apoptosis rate = (no. of apoptotic cells/no. of total cells in blastocyst) × 100.
